# Does virtual reality increase the emotional well‐being benefits of online psychoeducation? Preliminary results in informal caregivers of people with Alzheimer’s disease

**DOI:** 10.1002/alz.086791

**Published:** 2025-01-09

**Authors:** Cristina Festari, Claudio Singh Solorzano, Maria Gattuso, Cristina Bonomini, Sandra Rosini, Clarissa Ferrari, Orazio Zanetti, Michela Pievani, Francesca Morganti

**Affiliations:** ^1^ IRCCS Istituto Centro San Giovanni Di Dio Fatebenefratelli, Brescia Italy; ^2^ University of Bergamo, Bergamo Italy; ^3^ IRCCS Istituto Centro San Giovanni di Dio Fatebenefratelli, Brescia., Brescia Italy; ^4^ Instituto Ospedaliero Fondazione Poliambulanza, Brescia, Italy, Brescia Italy; ^5^ IRCCS Centro San Giovanni di Dio Fatebenefratelli, Brescia Italy; ^6^ Universita Degli Studi Di Bergamo, Bergamo Italy

## Abstract

**Background:**

Increasing findings have proven that virtual reality (VR) is a promising approach for improving knowledge, self‐efficacy, and empathy in educational programs (Dhar, DigitHealth. 2023). The purpose of an ongoing randomised clinical trial is to enhance mental wellbeing of dementia patients' informal caregivers (iCGs) by including a VR‐based empathy training into an online psychoeducation program.

**Method:**

60 iCGs of mild‐to‐moderate patients with Alzheimer’s disease were randomized in an online 6‐week psychoeducational program (control arm) or in the same psychoeducational program integrated with VR (experimental arm). VR consisted of 360‐degree videos and allows caregivers to experience dementia symptoms from the patient’s perspective. Before and after the intervention, iCGs completed validate scales to assess burden (ZBI), perceived stress (PSS), anxiety (STAI‐Y1&2) and perceived self‐efficacy for caregiving tasks (RSCSE). Pre‐post differences were tested with paired t‐tests.

**Result:**

27 iCGs assigned to the control arm (mean age = 56; female: 82%; children: 63%) and 29 to experimental arm (mean age = 54; female: 79%; children: 68%) concluded the intervention. At baseline, iCGs show similar level of burden (ZBI: t(54) = ‐0.793, p = 0.431), stress (PPS: t(54) = ‐0.483, *p* = 0.631), anxiety (STAI‐Y1: t(54) = ‐0.197, p = 0.845; STAI‐Y2; t(54) = 0.363, p = 0.718). The VR‐based intervention decreased levels of burden (ZBI: t(28) = 2.154; p = 0.040; Cohen’s d = 0.400), stress (PPS: t(28) = 2.177, *p* = 0.038; Cohen’s d = 0.404), state anxiety (STAI‐Y1 = t(18) = 2.400, p = 0.23, Cohen’s d = 0.446) and increase two domains of caregiving self‐efficacy: responding to disruptive patient behaviors (RSCSE‐RDB: t(28) = ‐2.358, *p* = 0.026; Cohen’s d = 0.438), and controlling upsetting thoughts (RSCSE‐CUT: t(28) = ‐2.126, *p* = 0.042; Cohen’s d = 0.395). In the control arm, changes due to intervention did not reach statistical significance.

**Conclusion:**

These preliminary results support the feasibility and effectiveness of psychoeducation program integrated with VR in improving perceived self‐efficacy in managing an AD patient and decreasing distress in iCGs. These results should be confirmed in larger samples.

